# Healthy Ageing in Malaysia by 2030: Needs, Challenges and Future Directions

**DOI:** 10.21315/mjms2024.31.4.1

**Published:** 2024-08-27

**Authors:** Jafri Malin Abdullah, Amin Ismail, Muhamad Saiful Bahri Yusoff

**Affiliations:** 1 Head, Medical and Health Science Cluster, Majlis Profesor Negara, Selangor, Malaysia; 2 Deputy Head, Medical and Health Science Cluster, Majlis Profesor Negara, Selangor, Malaysia; 3 Secretary, Medical and Health Science Cluster, Majlis Profesor Negara, Selangor, Malaysia; 4 Department of Neurosciences and Brain Behaviour Cluster, School of Medical Sciences, Universiti Sains Malaysia, Kelantan, Malaysia; 5 Department of Nutrition, Faculty Medicine and Health Sciences, Universiti Putra Malaysia, Selangor, Malaysia; 6 Department of Medical Education, School of Medical Sciences, Universiti Sains Malaysia, Kelantan, Malaysia; 7 Center for the Development of Academic Excellence, Universiti Sains Malaysia, Pulau Pinang, Malaysia

**Keywords:** healthy ageing, geriatrics, health policy, health promotion, social support, Malaysia, ageing

## Abstract

This Editorial addresses the critical need for developing a healthy ageing society in Malaysia by 2030. With the country’s elderly population projected to increase significantly, the article explores current challenges, including healthcare disparities, a shortage of geriatric specialists and malnutrition. It evaluates existing policies and highlights successful international and local initiatives, suggesting specific recommendations to improve healthcare infrastructure, healthy ageing support and technological integration. Emphasising the importance of engaging private sectors, non-governmental organisations (NGOs) and community groups, this Editorial calls for a collaborative approach to address the economic and cultural aspects of ageing. This comprehensive strategy aims to ensure a resilient, healthy and inclusive environment for Malaysia’s ageing population by 2030.

## Introduction

An aging population is defined as one in which those aged 60 years old and above make up at least 15% of the total population. The increment of elderly population (60 years old and above) is a global phenomenon in the high- and middle-income countries. The agenda for Sustainable Development Goals is just 6 years away in 2030 when the 15% of Malaysia population are expected to be aged 60 years old and above.

Based on national and international guiding policies and documents, several plans and strategies have been developed by government agencies for promoting a healthy and wellbeing ageing society in Malaysia. To support a healthy ageing society, the government of Malaysia through the Ministry of Women, Family and Community Development has developed a National Policy for the Elderly (Dasar Warga Tua Negara) in 1995 and in 2011, it was improved and renamed to National Policy for Older Persons (Dasar Warga Emas Negara [DWEN]). The purpose of this policy is to empower the individual, family and community by providing age-friendly supports and environments. The Senior Citizens Activity Centre (PAWE) was established under this policy and strategically partnership with other government agencies and non-governmental organisations (NGOs) in the country. It serves as a platform for elderly people to carry out and participate in activities organised by the centre. Through these activities, they will socialise within the community and it will prevent loneliness and depression among them.

There are policies, guidelines and procedures to combat poor health and well-being of elderly people, one of policies is National Health Policy for Older Persons was established in 1995 under the Ministry of Health Malaysia. The purpose of this policy is to ensure healthy ageing and improve the quality of life by empowering the elderly, their families and communities with support systems. In Malaysia, October 1st every year is established as the National Day of Older Persons since 1992 in line with the resolution No. 45/106 made at the United Nations General Assembly on 14 December 1990. This celebration is to recognise the invaluable knowledge and contributions of older persons to the community and country.

Although several plans and strategies have been embarked on to promote the elderly people to be healthier and improve their quality of life, active involvement of national stakeholders to support healthy ageing is necessary. Furthermore, it is important to actively involve older people in decisions on ageing to capture their need. The lack of resources in the country such as trained caregivers and aged-friendly facilities, especially in rural areas, need to be addressed urgently.

### Demographic and Health Trends in Malaysia

The demographic trend had shifted with the percentage of the elderly people (aged 60 years old and above) increased over the past two decades in ASEAN countries (Table 1). During the same period, the highest percentage of elderly population in Singapore from 7.2% in 2000 to 16.6% in 2020, while Cambodia exhibited the smallest increase from 3.5% in 2000 to 4.7% in 2020. The trends reflect longer and healthier lives.

Over the past two decades, Malaysia’s demographic structure had shifted towards an ageing population, with the proportion of the elderly individuals increasing from 4% in 2000 to 7.2% in 2022. In addition, the productive working-age population (20 years old–59 years old) increased from 49.9% in 2000 to 57.2% in 2022 (Table 1). Therefore, the old-age dependency ratio (the number of elderly people, aged 65 years old and above per 100 productive working-age population, 20-year-old–59-year-old) in Malaysia increased by 4.6 (from 8.0 in 2000 to 12.6 in 2022).

**Figure 1:**
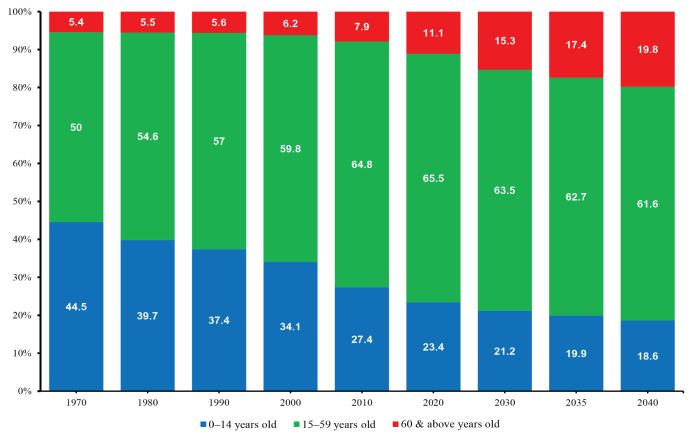
Percentage of the population in Malaysia by age-group (1970–2020) and projected population (2030–2040) Source: Department of Statistics Malaysia (2)

**Figure 2:**
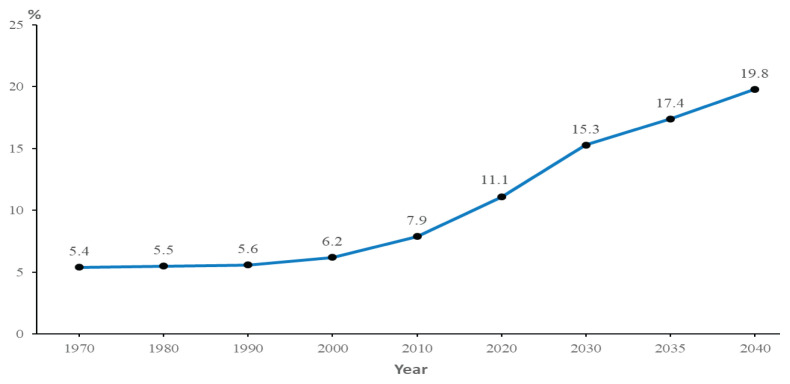
Percentage of elderly people in Malaysia (60 years old and above) and projected population (2030–2040) Source: Department of Statistics Malaysia

The demographic transition due to longer lives and smaller family members contributes to an ageing population. The composition of Malaysian aged 60 years old and over has increased from 6.2% in 2000 to 11.1% in 2020 to approximately 3.7 million individuals (Figure 1). It is projected that elderly people (60 years old and above) will increase to 15.3% in 2030 to approximately 5.8 million. In 2040, projection population of Malaysia is about 41 million with estimation of elderly people about 8.2 million individuals (Figure 2) (3). Therefore, the importance of health and well-being of individuals is crucial to meet the needs of a rapidly ageing society. Good nutrition, healthier lifestyle, family and government support, and social networking are important to create future generations of older people in Malaysia. According to the population data produced by the Department of Statistics (4), Johor, Kelantan, Pulau Pinang, Sabah and Sarawak have more than 7% of their population aged 65 years old and above. There are several districts that have the highest elderly population, more than 12%.

Good nutrition is essential for elderly. It helps them to meet their nutrients requirement for maintaining health and well-being. Ageing increases the risk of malnutrition. Malnutrition among the Malaysian elderly is growing concern. Based on the recent findings from the National Health of Mobility Survey (NHMS) 2018, the prevalence of malnutrition (7.3%) or at-risk of malnutrition (23.5%) among the elderly in Malaysia (5). The study found that food insecurity is one of factors contribute to the risk of malnutrition among the Malaysian elderly in rural area. To prevent and treat malnutrition, it is important for the elderly people to eat a balanced and variety of diet that meets their nutritional needs. There are many reasons why elderly may experience food insecurity and therefore may not be able to meet the nutritional requirements. Ensuring nutritional security may involve a multistakeholder approach to provide education on nutrient needs, economics, food preparation and other aspects to tackle the issues of malnutrition among elderly.

### Global Perspectives on Healthy Ageing

In 2020, the United Nations (UN) declared 2021–2030 as the Decade of Healthy Ageing, is aligned with the 2030 Agenda for Sustainable Development Goals. The Decade is a global initiative and collaboration between all Member States to improve the lives of older people, their families and communities. Under the World Health Organization (WHO), the Decade has focused four areas: i) Combatting Ageism—aims to shift perceptions and actions regarding age and ageing; ii) Age-friendly Environments—ensure that communities foster the abilities of older people; iii) Integrated Care—provides health and social services for older people and iv) Long-term Care—provides access for older people who need it (6). Governments, national and international organisations, civil society, private sector, academia, community groups and the media are encouraged to actively participate in achieving the Decade’s goals through a Healthy Ageing Collaborative (HAC) platform. This platform is aimed to promote and strengthen different sectoral and stakeholders’ collaboration to foster healthy ageing especially the implementation of the UN Decade of Healthy Ageing 2021–2030.

A report on the progress of the UN Decade of Healthy Ageing from 2021 to middle of 2023 implementation was presented at the UN General Assembly in 2023. In the first phase, national progress indicators developed for the WHO Global Strategies and Action Plan on Ageing and Health (2016–2020) were used to monitor the progress. These indicators were based on four areas with the aim to assess the extent progress made in the implementation of the Decade of Healthy Ageing. The report found that member countries have developed new policies, strategies and frameworks, established new mechanisms and strengthened data collection on health ageing. In 2022, approximately 82% of countries have enacted national legislation to address age-based discrimination, a significant increase from 60% in 2020. Additionally, 77% of member countries now have national programs to support age-friendly communities, up from 52% in 2020. Similarly, policies on long-term care for elderly people are now present in 78% of countries, compared to 67% in 2020. Furthermore, 71% of countries have implemented policies to support the comprehensive evaluation of health and social needs for elderly people, a substantial rise from 48% in 2020 (7). While these improvements in the four key areas are commendable, more efforts are needed to fully address the challenges of an ageing population.

Data from the report showed that Singapore has undertaken intergenerational activities to address ageism and the work of an Ombudsman for elderly people in Finland. There are actions in many countries (USA, India, Japan, South Africa and Ireland) to support aged-friendly cities and communities. There are countries reported that integrated care to meet the needs of elderly people for comprehensive health and care, however, challenges ahead are human resources constraints. Several initiatives have been conducted at national and regional levels to strengthen long-term care of elderly people (8).

Two cities (Penang Island, Pulau Pinang and Sibu, Sarawak) in Malaysia were recently recognised by the WHO Age-Friendly Cities and Communities network as a global community that aiming to foster age-friendly community development to promote and strengthen healthy ageing (9). Other cities in Malaysia are Ipoh and Taiping in Perak (10).

Malaysia has established policies, guidelines and strategies in line with the declaration of the UN 2021–2030 as the Decade of Healthy Ageing. It will improve and strengthen the healthy ageing and well-being of elderly people. There are growing number of elderly facilities in Malaysia, however, many elderly persons in the rural areas are still not aware the availability of these facilities. Limited resources are critical to promote healthy ageing and well-being. Therefore, more need to be done before silver tsunami coming to our country.

### Challenges to Healthy Ageing in Malaysia

Rapid urbanisation in Malaysia has led to healthcare infrastructure development primarily in urban areas, neglecting the healthcare needs of elderly individuals in rural and remote regions. Consequently, healthcare facilities in Malaysia may lack the capacity to accommodate the growing elderly population, resulting in long waiting times for appointments and treatments, thereby delaying care (11, 12). As Malaysia progresses towards becoming an ageing nation, it is crucial to address issues related to facilities, transport systems and the provision of elderly-friendly health services. The situation is particularly challenging for those who are single and must navigate these challenges alone, a reality that will become more pressing as 15% of Malaysia’s population is predicted to be over 65 years old within 6 years (13). To ensure the well-being of senior citizens, an increased number of physicians are required to provide geriatric care in both government and private hospitals (14).

Malaysia’s healthcare system is still developing its geriatric care infrastructure, facing a shortage of specialised healthcare professionals trained in geriatric medicine, which results in a lack of tailored care for older adults. According to the former Minister of Family, Women and Community Development, Dato’ Seri Rohani Abdul Karim, Malaysia needs at least 700 geriatricians and specialists to meet the demands of the increasing elderly population, emphasising the importance of a robust social welfare system to ensure a prosperous and healthy life for the elderly (15, 16). Public healthcare facilities in Malaysia often experience long waiting times and overcrowding, posing challenges for elderly patients who require more time and attention from healthcare providers, leading to delays in diagnosis and treatment and impacting the overall health and well-being of older adults (17).

Previous work environments may expose individuals to occupational hazards, leading to long-term health issues. Workers in industries like agriculture, manufacturing or construction may face higher risks of respiratory problems, musculoskeletal disorders or injuries, impacting their quality of life in older age (18). Access to nutritious food is essential for healthy aging. Food insecurity, limited access to fresh produce and unhealthy dietary habits can contribute to malnutrition, obesity, and diet-related diseases among older populations (19).

Older adults from lower-income households may struggle to afford medications, preventive screenings or proper nutrition, leading to higher rates of chronic diseases and premature aging. Education plays a crucial role in understanding health-related information and adopting healthy behaviours. Older adults with lower levels of education may have less awareness of preventive measures, leading to higher risks of health problems (20). Strong social networks and support systems are essential for healthy aging. Older adults who lack social connections or live alone may experience loneliness, depression and stress, which can negatively affect their physical and mental well-being (15).

In Malaysian culture, there may be a stigma attached to aging, where older individuals are sometimes viewed as less capable or less valuable than younger generations. This stigma can lead to discrimination in various aspects of life such as employment opportunities and access to healthcare. Cultural and societal factors, such as language barriers or distrust of healthcare providers, may further exacerbate these disparities (21).

Cultural norms and beliefs about health and illness may influence older individuals’ willingness to seek medical attention or adopt healthy behaviours. Traditional remedies and alternative medicine practices may be favoured over Western medical treatments, leading to delays in seeking appropriate care (22).

### Strategies for Promoting Healthy Ageing in Malaysia

Malaysia should develop comprehensive policies specifically aimed at promoting healthy aging. These policies should cover areas such as healthcare, social services, employment, housing, and transportation tailored to the needs of older adults (23). A centre known as the PAWE was established under DWEN. PAWE was established in collaboration with several NGOs and it needs to be improved to ensure the care of the elders while they family members are out working. PAWE is also a place where the elders can be self-reliant and interact with their elderly friends and the local community. Furthermore, the elders can gain knowledge and other benefits from the establishment of PAWE. For instance, PAWE serves as a community centre for the elders to socialise so they will not feel isolated or experience the empty nest syndrome. It is hoped that the government can establish more PAWE in rural areas so that children can bring their elderly parents in the morning to this centre and pick them up after work. Some of the activities carried out at PAWE are related to health, sports, recreation, therapy or rehabilitation, training/courses religion and volunteering services. PAWE was established to prevent loneliness and depression among the elders and prevent them from being sent to welfare centres. Health professionals are required to review current policies to improve the readiness of the population to care for the elders (15).

In 1995, Singapore passed the Maintenance of Parents Act into law. The Act aims to ensure the children take care their parents’ welfare by providing monthly expenses, food and clothing, as well as healthcare facilities. Those who disobey the law shall be fined. Also, Singapore has established an agency that provides accommodation for elderly parents. The agency provides free services to elderly parents who complain they have been neglected by their children. The purpose of this agency is to reiterate that if parents have raised their children, then the children should return the favour by shouldering the responsibility to support their parents without solely relying on the government to support and care for the elderly parents (15). The approach taken by Singapore should be adopted in Malaysia so that children will be more aware to care for their elderly parents and this will hinder the children from neglecting their responsibility since the number of cases reported in the local media concerning elderly parents being abandoned has increased. Therefore, it is hope Malaysia will enact a legislation to ensure children take care of their parents’ welfare rather than leave the elders alone to reduce the influx of elderly parents living in welfare homes and care centres elders (15).

Develop integrated care systems that coordinate healthcare services across various providers and settings, including hospitals, primary care clinics, community health centres and long-term care facilities. This ensures seamless transitions between different levels of care and improves continuity of care for older adults. Utilise telemedicine and remote monitoring technologies to provide healthcare services to elderly patients, especially those living in remote areas. This can help improve access to healthcare and facilitate early intervention for health issues. Carry out research in gerontology, geriatrics and gerontechnology disciplines. These areas of study could unlock new possibilities for healthy aging and drive an innovation in addressing the multidimensional challenges and opportunities of the ageing population. Research based on development, commercialisation and innovation needs to be developed in our country as a preparation for facing an ageing country and supporting healthy, active and productive ageing (13).

Develop age-friendly infrastructure and environments that support healthy aging, such as accessible healthcare facilities, pedestrian-friendly neighbourhoods and recreational spaces designed for older adults (15). Expand government subsidies and financial assistance programmes to make healthcare services more affordable for seniors, particularly for essential medical treatments, medications and assistive devices. Malaysia government can introduce or expand subsidies that are tied to income levels that can ensure that low-income seniors have access to necessary healthcare services without facing financial hardship. The other away is to implement cost-sharing reductions, such as lowering deductibles, co-payments and coinsurance for seniors with limited financial resources. This can help alleviate the financial burden on elderly individuals and their families (24). Enhance health insurance coverage for seniors, either through public healthcare schemes or private insurance plans, to ensure comprehensive coverage for medical expenses, including hospitalisation, specialist consultations, diagnostic tests and long-term care services.

Social care programmes ensure that elderly individuals have access to essential healthcare services. This includes regular check-ups, medication management and specialised care for chronic conditions prevalent in older adults. Financial Assistance such as Waqf Fund may offer financial aid to elderly individuals who are economically disadvantaged or do not have sufficient retirement savings. This assistance can help them meet their basic needs such as food, housing and utilities. Many elderly individuals require assistance with daily activities. Social care programmes provide training and support for caregivers, ensuring that they have the skills and resources necessary to provide quality care (25).

Strengthen community-based care services to support aging in place. This involves establishing community health centres, day care centres and homecare services to provide older adults with access to healthcare, social support and recreational activities within their communities (26).

Implement health promotion campaigns targeting seniors to raise awareness about healthy lifestyle choices, preventive healthcare measures and early detection of chronic diseases. Encourage regular health screenings, vaccinations and adherence to medication regimens. Provide specialised training and continuing education programmes for healthcare professionals, including doctors, nurses and allied health professionals, to enhance their knowledge and skills in geriatric care, age-related health issues and communication with elderly patients.

The lifestyle modifications can play a crucial role in healthy aging and the prevention of non-communicable diseases (NCDs). An adherence to healthy lifestyle elements, including physical activity, healthy diet, moderate alcohol consumption and not smoking, was associated with a longer life expectancy. The importance of nutritional consideration needs to be emphasise in promoting healthy aging and preventing NCDs for healthy aging and the reduction of age-related chronic diseases. Thus, tailored dietary interventions must be addressed with the specific nutritional requirements of the ageing population in the context of NCDs prevention (12). Many elders in the rural areas are not aware of the importance of undergoing medical check-ups to detect the early signs of any chronic diseases. Therefore, the rural community have to be exposed to better healthcare initiatives so that the elders living there can remain active and productive in their golden age (15).

### Technological Innovations and Opportunities

In the quest to support healthy ageing, technological innovations offer significant opportunities to enhance the quality of life for older adults in Malaysia. Leveraging digital health solutions, assistive technologies, and fostering research and development can address many challenges faced by the elderly, promoting independence and well-being (27).

The rise of digital health technologies offers numerous possibilities for improving healthcare accessibility and management for older adults (27). Telemedicine services enable elderly patients to consult with healthcare providers from home, reducing the need for travel and making healthcare more accessible, particularly for those in remote areas, while facilitating regular monitoring and early intervention for chronic conditions (28). Mobile health applications and wearable devices can monitor vital signs, remind patients to take medications, and track physical activity, empowering older adults to manage their health proactively and providing healthcare professionals with valuable data for personalised care (29). Additionally, comprehensive electronic health record (EHR) systems ensure that healthcare providers have access to accurate and up-to-date patient information, thereby improving the continuity and quality of care for older adults with complex health needs (30).

Assistive technology plays a crucial role in maintaining or improving an individual’s functioning in areas such as cognition, communication, hearing, mobility, self-care and vision, thereby enhancing health, well-being, inclusion and participation for older adults (31, 32). Innovations in mobility aids, such as advanced wheelchairs, walkers and scooters, help older adults maintain independence and mobility, with these devices becoming increasingly user-friendly and adaptable to various needs (32). Smart home technologies, including voice-activated assistants, automated lighting and emergency alert systems, create safer living environments and help manage daily tasks while providing emergency assistance, giving peace of mind to both seniors and their families (33). Additionally, advances in hearing aids and vision enhancement devices significantly improve the quality of life for older adults with sensory impairments, allowing them to stay connected and engaged with their communities (34).

Investing in research and development is crucial for driving innovations that address the unique needs of an ageing population (35). Supporting research on age-related diseases, geriatric care, and the impact of ageing on physical and mental health can lead to new treatments and interventions, with collaboration between academic institutions, healthcare providers, and the private sector accelerating these advancements (36). Encouraging the development of products tailored to the elderly, such as ergonomic furniture and adaptive clothing, can enhance comfort and usability, thereby improving the quality of life for older adults (37). Additionally, implementing pilot programmes and clinical trials to test new technologies and interventions in real-world settings can provide valuable insights and evidence for broader adoption, helping to identify best practices and refine solutions to meet the specific needs of Malaysia’s elderly population.

Embracing technological innovations and fostering a culture of research and development can transform the landscape of elderly care in Malaysia. These efforts require collaboration among government agencies, healthcare providers, technology companies and research institutions to ensure that the benefits of technological advancements are accessible to all older adults. By harnessing the power of technology, Malaysia can create an environment that supports healthy ageing, enabling older adults to live independently, safely and with dignity. The integration of digital health solutions, assistive technologies, and a robust research and development framework will play a pivotal role in achieving these goals, ultimately contributing to a healthier, more inclusive society.

### Role of Stakeholders

Promoting healthy ageing in Malaysia requires a concerted effort from multiple stakeholders, each playing a critical role in creating an environment that supports the well-being of older adults (36, 37). From government and healthcare providers to community organisations and the private sector, collaboration is key to addressing the multifaceted challenges of an ageing population (38).

The government plays a pivotal role in establishing the policy framework and allocating resources to support healthy ageing (39). Government bodies must develop and implement comprehensive policies that address the health, social and economic needs of older adults, integrating ageing issues into national health plans and social policies. Adequate funding for health and social services is essential, and the government should prioritise investments in healthcare infrastructure, social protection schemes and community support programmes to ensure that older adults have access to necessary services. Additionally, enacting laws to protect the rights of older adults, combat ageism and promote age-friendly environments is crucial, with government-led advocacy campaigns raising public awareness about the importance of healthy ageing and encouraging societal change.

Healthcare providers are at the frontline of delivering services that directly impact the health and well-being of older adults (40). They must be trained in geriatric care to address the unique health needs of older adults, including managing chronic diseases, providing preventive care and offering mental health support. Developing integrated healthcare models that coordinate medical, social and long-term care services ensures a holistic approach to elderly care, with multidisciplinary teams working together to provide comprehensive care plans for older adults. Additionally, healthcare providers can lead community-based health initiatives that promote healthy lifestyles, regular health screenings and disease prevention among older adults.

Community organisations and NGOs play a crucial role in supporting and creating inclusive environments for older adults (41). They establish social support networks that offer companionship, recreational activities and volunteer opportunities, helping to reduce loneliness and social isolation. Additionally, NGOs advocate for the rights and needs of older adults, raise awareness about healthy aging, influence public policy, and educate communities on the value and potential of older adults. Furthermore, these organisations provide essential services, including homecare, transportation, educational programmes and legal assistance, ensuring that older adults have access to necessary resources.

The private sector has significant potential to drive innovation and create products and services tailored to the ageing population (37, 42). Businesses can focus on developing age-friendly products, such as assistive devices, health monitoring tools and ergonomic home furnishings, to improve the quality of life for older adults. Employers can also foster workplace inclusion by implementing policies that support older workers, including flexible work arrangements, lifelong learning opportunities and retirement planning services, to keep them active and engaged in the workforce. Additionally, companies can contribute through Corporate Social Responsibility (CSR) initiatives by funding health programmes, sponsoring community centers and offering volunteer services to assist ageing communities.

Research institutions and universities play a crucial role in advancing knowledge and developing evidence-based strategies for healthy ageing (43). They conduct research on ageing-related issues, such as the biology of ageing, social determinants of health and effective interventions, which informs policy and practice. Collaborative research with international partners can provide global perspectives on local challenges. Additionally, academic institutions offer specialised training programmes for healthcare professionals, social workers and caregivers to equip them with the necessary skills for supporting healthy ageing. Researchers also analyse the impact of existing policies and programmes on older adults, offering valuable insights and recommendations for improvements.

By leveraging the strengths and resources of all stakeholders, Malaysia can build a robust support system for its ageing population. Collaboration and coordination among government agencies, healthcare providers, community organisations, the private sector and academia are essential to creating a society where older adults can thrive, enjoying good health, social inclusion and economic security (43). Together, these efforts can ensure that the elderly are valued, supported, and empowered to lead fulfilling lives.

## Conclusion

In summary, Malaysia is at a pivotal juncture in addressing the needs of its ageing population. As the proportion of older adults rises, adopting a comprehensive approach that promotes healthy ageing and enhances seniors’ quality of life is imperative. The challenges to healthy ageing in Malaysia are multifaceted, requiring coordinated efforts to strengthen healthcare services, promote social protection and foster inclusive communities. Technological innovations and advancements in digital health solutions and geriatric research present significant opportunities. To achieve a healthy ageing society, it is crucial to enhance the quality of life, ensure dignity and enable active participation for older adults by shifting societal attitudes towards ageing. The journey towards a healthy ageing society in Malaysia is a collective responsibility that requires commitment, innovation and compassion, benefiting not only the elderly but also the overall well-being and prosperity of the nation.

## Figures and Tables

**Table 1:** ASEAN’s demographic trends from 2000–2022

Country	Year	0–19 (years old)	20–59 (years old)	60–64 (years old)	≥ 65 (years old)

		%	%	%	%
Brunei Darussalam	2000	38.8	57.0	1.4	2.8
	2022	27.8	61.7	4.1	6.4

Cambodia	2000	54.1	40.4	2.0	3.5
	2022	40.4	52.3	2.6	4.7

Indonesia	2000	47.5	46.7	2.0	3.8
	2022	38.9	51.9	3.2	6.0

Lao People’s Democratic Republic	2000	42.5	49.3	2.1	6.1
	2022	30.9	56.3	4.5	8.3

Malaysia	2000	44.0	49.9	2.1	4.0
	2022	31.7	57.2	3.9	7.2

Myanmar	2000	38.1	53.0	3.2	5.7
	2022	35.4	53.6	4.0	7.0

Philippines	2000	47.5	46.7	2.0	3.8
	2022	38.9	51.9	3.2	6.0

Singapore	2000	28.4	61.0	3.4	7.2
	2022	19.4	56.9	7.1	16.6

Thailand	2000	27.0	59.6	4.3	9.1
	2022	22.6	58.0	6.5	12.9

Vietnam	2000	42.5	49.3	2.1	6.1
	2022	30.9	56.3	4.5	8.3

ASEAN	2000	40.8	51.4	2.5	5.3
	2022	32.5	55.8	4.2	7.5

Source: ASEAN Statistic Brief, Vol III, 15 December 2023 (1)
